# Academy of Stomatology

**Published:** 1895-05

**Authors:** 


### ACADEMY OF STOMATOLOGY.

The meeting of the Academy took place March 12, 1895. President Dr. Jack in the chair. A paper was read by Dr. Harrison Allen, entitled "Diseases of the Maxillary Sinus."

(For Dr. Allen's paper, see page 261.)

### DISCUSSION.

*Dr. Essig.*—I have had very little experience in the treatment of cases of diseases of the maxillary sinus. Within the last two or three years I have had two of some interest. One was a gentleman, the origin of whose trouble I was ignorant of. He came to me from a doctor in New York who had commenced treatment and had removed the first molar tooth, and had made an opening into the sinus and had introduced a silver tube. The discharge in the case was very copious.

When the gentleman came to Philadelphia to reside he found some difficulty in continuing the treatment. He would be sometimes three days in Philadelphia and then on the road for three months, and during that time the sinus was not irrigated or washed out, and the remedy was not applied.

I found he was not only intelligent, but quite handy in the use of instruments, and I obtained for him one of the little India-rubber syringes, such as we use every day in our practice, with a metallic point, and he took with him Seiler's tablets; with one of these dissolved in water, by the assistance of the little syringe the sinus was washed every day. When he was in Philadelphia he would always come to see me, and I occasionally removed the tube and renewed the ligatures attached to the neighboring teeth.

Finally, he was absent for three months, and when he came back I found the tube had been removed, the fistula had closed, and the sinus was quite well. There was no great difficulty in producing a cure so long as he could receive proper treatment.

Another case which came to my notice very recently was the result of an alveolar abscess of the first molar tooth which had been applied with a Richmond crown. In that case I felt very anxious, and was very much afraid the patient would have a serious time. Finally, the abscess was ready to be opened with a lance; after that the trouble subsided, and is now quite well. The discharge from the maxillary sinus into the nasal cavity of the mouth was quite large. After one of the visits she made some remark that arrested my steps before I reached the door, and I found her leaning over the banister. She said, "Just as I spoke to you an immense amount of fluid seems to have run out of my nose." Of course, I felt anxious about the case, and thought that if within a few days the symptoms did not subside it would be necessary to remove the first molar tooth and establish an opening; and I found that after the abscess was removed the swelling disappeared, the soreness subsided, and there was no further discharge or trouble with the sinus.

I had received the idea, probably, from Dr. Gross's teaching, or from his System of Surgery, that cases of trouble in the maxillary sinus were not only exceedingly serious, but very apt to take a malignant character; and I was very much afraid that the latter case would not get well without further and perhaps surgical treatment other than removal of the tooth and opening into the sinus; but I was very much gratified to learn that all the symptoms had disappeared.

My experience is limited, and I have often thought it curious that in more than twenty years of active practice I have met with but those two cases of trouble of antral inflammation. I do not hesitate to say they are exceedingly rare, in our practice at any rate. I know of no other than the two I have mentioned in all that time, out of a fairly large practice, where I have found any trouble or where it was necessary to treat. I know of no patient who has gone into the hands of a general practitioner or surgeon.

I would be very glad to hear from other members of the profession. My experience has certainly been very limited.

*Dr. Cryer.*—It gives me the greatest pleasure to have this opportunity of listening to Dr. Allen, not only for the knowledge I have received this evening, but also because he was my first teacher of the anatomy and surgery of the maxillary bones. He was also the first one whom I saw use an engine for bone surgery. This was while I was a student, some time in 1875; and, if I remember correctly, the operation was for the removal of an osteophyte under the upper lip near the symphysis of the maxillary bones.

It has been my very great pleasure to see Dr. Allen operate on the superior maxillary bones and their associates many times, and am indebted to him for a great part of whatever knowledge of the subject I may possess.

I have a case now that may be interesting, as I have been unable to stop the flow of pus. The patient came to me about two months ago. The first molar had been extracted, and in looking at the parts they appeared to be in a healthy condition, but when the mouth was thrown widely open, the pus would exude from where the tooth had been extracted, and in opening and closing the mouth frequently it appeared as though it pumped the pus out; the posterior wall of the antrum had broken down by caries or other lesion. In treating this antrum by different fluids, using peroxide of hydrogen, it was impossible to get any of the fluid forced into the nasal chamber, from which I concluded that that opening between the antrum and nasal cavity was closed; it was difficult to syringe the antrum from the small opening; thinking best to open it wider, the second molar was extracted, and by the use of a small surgical bur, the opening was made so that a syringe could be used of considerable size, and the cavity might be flooded or irrigated. It responded to treatment for a short time fairly well, but I noticed to-night there is still pus in the cavity, not having had it washed during the last two days. You can see a pulsation at the opening.

We have with us Dr. Gleason, who is interested in diseases of the antrum, and I would be very glad to hear from him.

*Dr. Gleason.*—My experience has been very limited in diseases of the maxillary sinus, so that it hardly seems worth while for me to say anything about it. I think I have had only five cases. I cannot remember more than that number. Of these, three of them were from causes outside of the teeth. If I am not mistaken, Dr. Bosworth, the well-known writer upon such diseases, states that diseased teeth are regarded as mostly the cause of maxillary sinus, yet rhinologists will take a different view of the subject, and consider that lesions of the maxillary sinus are more often of the intra-nasal region.

In the first of these cases that came under my observation the trouble was acute in its nature, being the result of a cold: a rheumatic individual had caught a cold and got well inside of ten days; at least, it was not over two weeks before the symptoms of the disease of the maxillary sinus ceased.

In the second case, the disease was apparently the result of nasal polypus, with symptoms of empyema of the parts from time to time. In the mean time she consulted her dentist, and during that time the disease of the maxillary sinus developed, apparently, and was treated by the dentist. She was not cured at the time I saw her.

In the third case the anterior turbinated bone had been lost as a result of syphilis, so that it was possible to look from the nose into the maxillary sinus and see what came from it. During one or two attacks of cold the nasal membrane became very much congested. It lasted for a length of time and finally subsided.

On another occasion, inflammation of the sinus was followed by a somewhat copious discharge of serum. In the third and fourth of these cases it was the result of disease of the incisor teeth, and involved the bone above so that the maxillary bone became necrosed, the result of the extension of the inflammation of the sinus. The right side was involved, and there was purulent inflammation of the cavity.

A temporary relief of the affection was brought about somewhat promptly by washing it with peroxide of hydrogen, a fifteen-volume solution, and afterwards following that up with a four-grain solution of nitrate of silver to the ounce of water,—four grains to the ounce. A solution of that strength is so strong that often if care is not taken it will irritate the mucous membrane greatly before washing the nasal parts.

There were one or two relapses, however, in this case, but an ultimate cure of the affection was brought about.

*Dr. Currie.*—I would like to ask Dr. Allen in regard to a case I had. It was the second week after I graduated. I went into a rural district, and I think it was the third or fourth call. An old lady, of about sixty years of age, who said she had something the matter with her "gooms," and wanted me to look at them. There were no teeth from the cuspid tooth back, and it did not look as if there had been for years; but she insisted there were some roots there and that they hurt her. She had been to the doctors a good deal and they told her she was only nervous.

I took a lance and passed it through the gum into the antrum, and there was a discharge of most offensive pus. I treated her, and before the end of the second week she came in and complained very much of the other side of the face, which was quite swollen. I opened that, but it was three months before the parts healed.

The question I would like to ask is, Would you place this in the five classes? I was not able to locate the cause, unless it had been from catarrhal trouble. She had been treated some years before for this disease. I saw her several months afterwards and there was no discharge. I thought perhaps it was not from catarrhal trouble after all.

*Dr. Darby.*—Dr. Allen said that these diseases are more common than we imagine. After making that remark I recalled the instance of a surgeon of this city who called upon me twice if not three times during the past twenty years to have me examine the teeth carefully under the antrum to ascertain whether there was any dental cause which could give rise to abscess of the sinus. I found on each occasion that the teeth were perfectly sound, and I had no reason to believe that this abscess or this condition came from the teeth. It came each time after a severe cold and catarrhal condition, and he complained not only of great fulness in the mucous passages of the head and masticatory muscles, but also complained of a discharge when lying on the left side, a discharge from the nostril; from this he suffered a good deal of pain.

In a few weeks after the catarrhal condition passed away, the pain ceased, and he had no return of it until another violent cold. I have seen him three times in twenty years with just that condition, so I presume there is a catarrhal condition of the membrane lining the antrum, which is not dissimilar from that which we have in the nasal passages. Am I right?

*Dr. Allen.*—The discussion has been very instructive and several points have been brought forward which are new to me. I have not had the experience that Dr. Gleason gives. I presume from what Dr. Darby said that he has recognized this transmission of irritation from the nasal chamber to the nose in a form by which he was enabled to diagnose the inflammation of the sinus. It only goes to show how narrow one's experience is. I have now, and I suppose all of us have cases come to us, sometimes several cases, and then they will go away and you will never see anything of them. It may be in the order of causes I should say that dental causes are more frequent than others,—Dr. Gleason would evidently not agree with me there. Of course, I give my conclusions only from my own experience. For my own part I would not make a diagnosis without illumination.

The case of the old lady is entirely new to me. I have seen plenty of cases in which the bones were so thin that the lance would have gone through the slight barrier between the gum-line and the anterior sinus, but for an old person to have pyæmia is a very interesting case, and the opening with the lance is, I believe, unique. I think the case ought to be reported.

Dr. Essig states that he has had but three cases in a practice of twenty years. I think a post-mortem examination would show it occurs more frequently than that. In the University of Pennsylvania I took all the refuse of the dissecting-room and had the heads separated and prepared for study. They were macerated and afterwards boiled, and out of twenty-five cases I found two in which there was a cheesy appearance of the maxillary sinus, which in that proportion would be eight in a hundred.

Now it is probable that in the lower classes we may see it more than in the higher classes. The cases are not, perhaps, so likely to be found in private practice as in the dissecting-room.

*Dr. Essig.*—Dr. Allen's suggestion that cases of that kind are not so likely to be seen in private practice as in a dissecting-room is probably true. I think a practice among the better class of people that is kept well in hand is not the very best place to observe other than our ordinary dental operations.

Some time ago, to illustrate, I wanted two or three samples of Hutchinson's teeth. I had not seen a Hutchinson tooth in my own practice in twenty years, and I had to ask Dr. Risley to look out for them among his clinical patients. In the course of a few days he was able to send me quite a number of little patients with typical Hutchinson teeth, and I was able to get an impression of them so that I procured quite a collection. In my own practice I would never have been able to secure them.

In these cases of antrum troubles, I would like to hear the experiences of my colleagues and what their experience has been. If I were asked I should say these cases are exceedingly rare.

*Dr. Burchard.*—In all the text-books, including that of Dr. Allen, in the description of the antrum it is said that the roots of the molars frequently perforate its floor. I want to know whether there can be any accurate anatomical description given of the covering of the apex of the root? That is, have preparations been made, showing the histological relations of the root-covering in such cases?

*Dr. Allen.*—I do not know.

*Dr. Burchard.*—In using peroxide of hydrogen for irrigation I have neutralized the acid in it with peroxide of sodium. It may be alkalinized and at the same time the volume of oxygen may be increased.

*Dr. Allen.*—I am glad to know that.

*Dr. Gleason.*—My experience has hardly been sufficient to base a rule upon, but Dr. Cryer has some at present, and could give more information on the subject.

*Dr. Jack.*—In my experience of forty-one years I have had but one case, with which Dr. Allen is familiar.

Dr. Cryer stated, in reply to Dr. Gleason, that the cases he has in hand at the hospital are cases coming from different parts of the State and outside of the State, so that it is not a fair average to take as a general class of patients. At present there are three reporting to the hospital with antrum troubles.

Meeting adjourned.

